# Comparison of clinical and radiological outcomes of local morselized bone grafts and structural iliac bone grafts in the treatment of lumbar tuberculosis with posterior-only surgery

**DOI:** 10.1186/s12893-022-01638-4

**Published:** 2022-05-14

**Authors:** Shuang Xu, Shuai Zhang, Gaoju Wang, Jin Yang, Yueming Song, Qing Wang

**Affiliations:** 1 Department of Orthopedics, Orthopedic Research Institute, West China Hospital, Sichuan University, Chengdu, 646000 Sichuan Province China; 2 Department of Orthopaedic Surgery, The Affiliated Hospital of Southwest Medical University, No. 25 of TaiPing Road, Luzhou, 646000 Sichuan Province China

**Keywords:** Lumbar spinal tuberculosis, Posterior approach, Morselized bone, Iliac bone, Surgery

## Abstract

**Background:**

Many surgeons have reported results similar to those of anterior debridement and bone grafting in treating spinal tuberculosis in the lumbar region using only a posterior approach. However, there is still no consensus regarding bone graft methods. This study aims to compare the clinical and radiological outcomes of morselized versus structural iliac bone grafts in the treatment of lumbar tuberculosis via one-stage posterior surgery.

**Methods:**

A retrospective study was performed with 82 patients with lumbar tuberculosis who had undergone posterior-only debridement, bone grafting, and instrumentation between January 2014 and June 2018. Morselized bone grafts were used in 43 patients, whereas structural iliac bone grafts were used in 39 patients. The clinical data and imaging results of the patients were compared between the two groups to evaluate the clinical effects of the two types of grafts.

**Results:**

The operation time, blood loss and hospital stay values in the morselized bone group were significantly lower than those in the structural iliac bone group (p < 0.05). No significant differences were observed with respect to erythrocyte sedimentation rate (ESR), C-reactive protein (CRP), Cobb angle, or improvement of neurological function between the two groups. The VAS pain scores for low back and leg pain decreased significantly after the operation (p < 0.05). However, postoperatively, the VAS score was higher in the structural iliac bone group than in the morselized bone group, and there was no significant difference at the last follow-up between the two groups (p > 0.05). Bone fusion was achieved in 41 patients (95%) in the morselized bone group and 38 patients (97%) in the structural iliac bone group. There was no significant difference between the fusion rates of the two groups (p > 0.05).

**Conclusion:**

The two graft techniques achieved comparable clinical outcomes in lumbar spinal tuberculosis treatment. However, the morselized bone graft was more beneficial in terms of reducing surgical trauma and postoperative complications.

## Introduction

The incidence of spinal tuberculosis is increasing in developing countries and often leads to severe kyphosis and permanent paralysis [1]. Chemotherapy is key for treating spinal tuberculosis. However, patients with spinal instability, nerve damage, and severe kyphosis often require surgical treatment. The main purposes of surgical treatment are radical debridement, nerve decompression, and spinal reconstruction to prevent or improve kyphosis.

The anterior approach provides direct access to the lesion site, facilitating lesion removal and reconstruction of the defect [2]. However, the anterior approach provides inadequate leverage for kyphosis correction. The combination of anterior and posterior approaches overcomes the limitations of the anterior approach and has been widely adopted with good results [3, 4]. It should be noted that the combined anterior and posterior approach increases the operation time and is not conducive to early patient recovery due to the large amount of trauma and high risk of complications. In recent years, many spine surgeons have performed one-stage posterior debridement, bone grafting, and instrumentation for spinal tuberculosis and achieved satisfactory clinical outcomes [5–7].

Radical debridement is vital for the surgical treatment of spinal tuberculosis, and bone grafting is necessary to restore the height of the vertebral body and reconstruct the stability of the spine [8, 9]. Although tricortical iliac bone grafts are the gold standard for spine reconstruction [10], many surgeons have recently reported satisfactory results when using morselized bone grafts to treat spinal tuberculosis [11–13]. Some scholars also believe that the structure of morselized bone is loose, the supporting force is weak, the correction of kyphosis is poor, and there is a risk of protrusion into the spinal canal [14]. Therefore, the difference in clinical efficacy between structural iliac bone and morselized bone in posterior surgery for lumbar tuberculous remains unclear.

This study aims to compare the clinical and radiological outcomes of using morselized bone versus structural iliac bone to treat lumbar tuberculosis via one-stage posterior surgery.

## Materials and methods

### Patient selection

This was a retrospective study and was approved by the ethics committee of the Affiliated Hospital of Southwest Medical University. Patients who underwent surgical treatment in our hospital for lumbar tuberculosis from January 2012 to December 2018 were retrospectively analysed.

Inclusion criteria: (1) The lesion involved one or two adjacent lumbar segments (L1–L5); (2) patients received debridement, autologous bone grafting, and instrumentation via a posterior-only approach; (3) a definitive TB diagnosis was obtained by pathological examination; and (4) a minimum of 2 years of follow-up was completed. Exclusion criteria: (1) multisegment lumbar tuberculosis; (2) other spinal diseases affecting the postoperative evaluation, such as adolescent scoliosis or ankylosing spondylitis; and (3) loss of follow-up data for any reason, including failure to follow up and death.

The primary diagnosis of tuberculosis was made according to the medical history, clinical examination, laboratory results, radiologic imaging, and drug response. A definitive diagnosis was made by histological examination and/or by polymerase chain reaction (PCR) analysis of tissue removed during surgery. The indications for surgery included intractable low back pain due to instability, progressive neurological dysfunction, kyphosis formation and negative responses to chemotherapy [11–13].

### Preoperative preparation

Conventional anti-TB treatment was administered 2–4 weeks before surgery. The HREZ chemotherapy regimen was administered, including isoniazid (300 mg/day), rifampicin (450 mg/day), ethambutol (750 mg/day), and pyrazinamide (750 mg/day) [6, 7]. Patients with malnutrition were given oral supplementation or parenteral nutrition to improve their preoperative condition.

### Surgical method

**Fig. 1:**
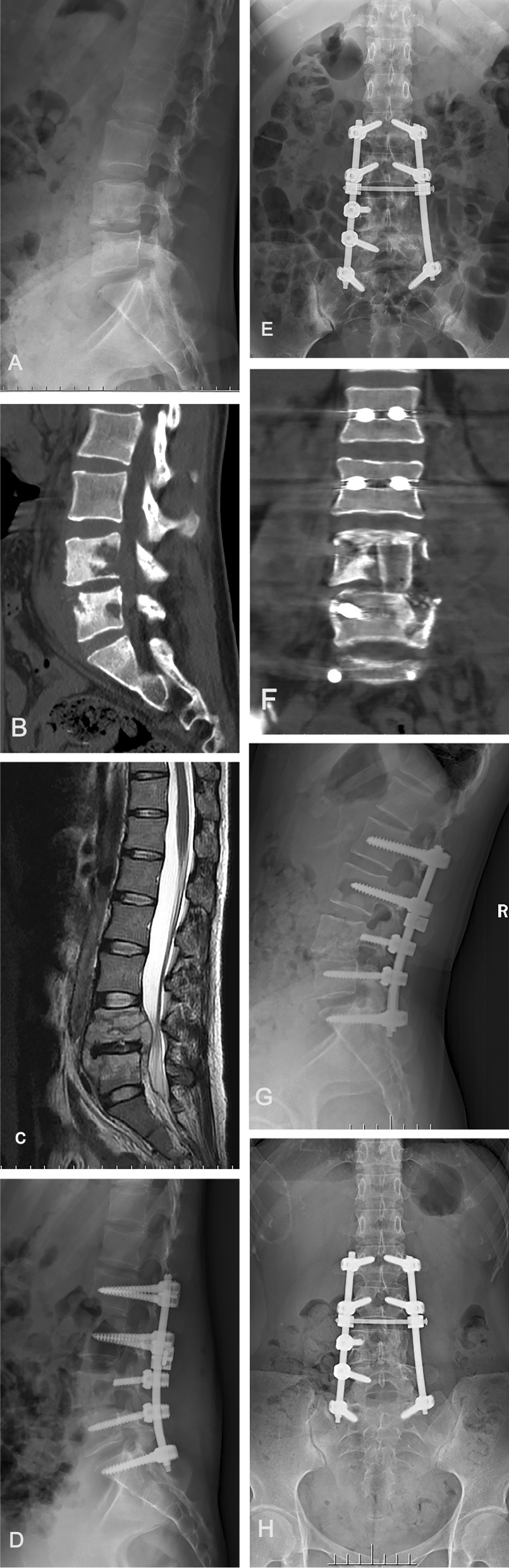
A 68-year-old male with L2–3 tuberculosis. **a** The lateral X-ray film before surgery demonstrated intervertebral stenosis between the L2–3. **b**, **c** Preoperative CT and MRI showed that L2–3 vertebral bone destruction. **d**–**f** Postoperative X-ray and CT of a patient who underwent posterior internal fixation from T12 to L5 and morselized bone graft at L2–3. **g**, **h** X-ray at 26 months postoperative showing bone fusion between L2 and L3, without signs of tuberculosis recurrence and hardware failure

**Fig. 2:**
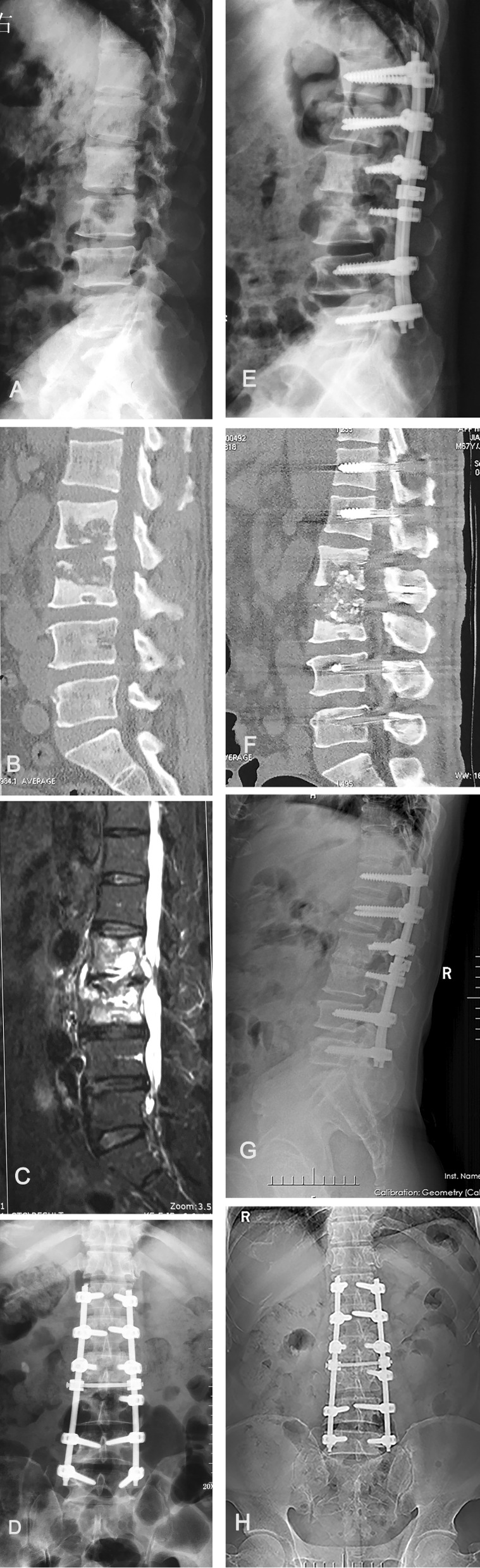
A 39-year-old female with L4–5 tuberculosis. **a** The lateral X-ray film before surgery demonstrated intervertebral stenosis between the L4–5. **b**, **c** Preoperative CT and MRI showed that L4–5 vertebral bone destruction with paravertebral abscess. **d**–**f** Postoperative X-ray and CT of a patient who patient underwent posterior internal fixation from L2 to S1 and structural iliac bone graft at L4–5. **g**, **h** X-ray at 24 months postoperative showing bone fusion between L4 and L5, without signs of tuberculosis recurrence

After administration of general endotracheal anaesthesia, patients were placed in the prone position. A posterior incision was made to expose the posterior spinal elements, including one or two vertebrae above and below the affected segments. Pedicle screws were inserted into one or two normal vertebrae above and below the affected vertebra, respectively. Short pedicle screws were implanted in the affected vertebra according to the characteristics and conditions of the damaged vertebral bone. Based on imaging data, the side with severe destruction and large abscess were selected as the operating side. After a temporary internal fixation rod was installed on the contralateral side, the lamina and articular processes were resected from the more severe side of the lesion, and the tuberculosis focus was exposed. If the lesion was extensive, the ipsilateral pedicle was removed to provide sufficient vision for debridement. Then, the sequestrum, caseous tissue, necrotic intervertebral disc, and paravertebral abscess were removed using different types of curets. After the lesion was cleared, the area was irrigated with a large amount of normal saline. If the contralateral lesion was not cleared satisfactorily or there was a large paravertebral abscess on the contralateral side, it was removed using the same method. After that, the local morselized bone harvested during surgery (morselized bone group) (Fig. 1) or structural iliac bone harvested from the iliac crest (structural iliac bone group) (Fig. 2) was implanted into the bone defect space to reconstruct the anterior column based on patient preference. Then, the prebent rod was installed, and the posterior screw system was properly pressurized or distracted to correct the kyphosis. Satisfactory internal fixation and correction of kyphotic deformities were confirmed by C-arm fluoroscopy. Then, a drainage tube was placed, and the incision was closed layer by layer.

### Postoperative care

The drainage tube was removed when the drainage volume was less than 50 ml/24 h [1]. Anti-tuberculosis chemotherapy, which was the same as the preoperative regimen, was continued for 3 months postoperatively, followed by a regimen of isoniazid, rifampicin, and ethambutol for another 12–15 months [6, 7]. Patients were allowed to ambulate with the support of a waist brace 7 days after the surgery.

### Follow-ups

All patients were examined at 3-month intervals in the 1st year and then every 6 months. Blood panels, the ESR, C-reactive protein (CRP), and hepatic and renal functions were checked during each follow-up. Plain films or CT scans were performed to investigate bony fusion. The bone graft fusion criteria reported by Bridwell et al. [15] were used to evaluate bone graft fusion, and the time of bone graft fusion was recorded. The American Spinal Injury Association (ASIA) criteria was used to evaluate preoperative and postoperative neurological function. Visual analogue scale (VAS) scores were used to assess the severity of pain. The preoperative and postoperative kyphosis angles were evaluated using the standard method for measuring the kyphosis angle [10]. Images parameters including Cobb angle and fusion status were independently measured by two senior spinal surgeons (SX. and JY). If there is any disagreement for graded data, they discuss together.

### Statistical analysis

All data were statistically analysed using SPSS 19.0 software (SPSS Inc., Chicago, IL). The normality of the data were tested by Shapiro–Wilk test before further statistical analysis. The interobserver agreement of images parameters was evaluated through the calculation of the kappa coefficient. The continuous variables were compared between the two groups by t test, and the classification data were analysed with the Mann–Whitney test or the Chi-square test. Paired t tests were used for intragroup comparisons. A P value < 0.05 was considered statistically significant.

## Results

### Patient information

There were 82 patients enrolled in this study, including 43 patients in the morselized bone group and 39 patients in the structural iliac bone group, according to the inclusion criteria. There was no significant difference in age, sex, follow-up time, lesion segment, paravertebral abscess, intraspinal abscess, or ASIA score (P > 0.05). The basic information of the patients in both groups is shown in Table 1.

**Table 1:** The preoperative basic information of patients in two groups

Measurements	Morselized bone group (43)	Structural iliac bone group (39)	P-value
Age (year)	47.4 ± 12.4	48.2 ± 12.0	0.912
Gender			
Male	23	22	0.756
Female	20	17	
Follow-up (month)	28.7 ± 4.3	30.1 ± 6.2	0.273
Segments			
L1–2	4	5	0.146
L2–3	13	11	
L3–4	12	14	
L4–5	12	8	
L5–S1	2	1	
Paravertebral abscess	30/43(69%)	28/39(71%)	0.840
Intraspinal abscess	12/43(27%)	9/39(23%)	0.617
ASIA scores			
C	2	1	0.666
D	4	6	
E	37	32	

### Clinical outcomes

The operation time, blood loss, and hospital stay in the morselized bone group were significantly less than those in the structural iliac bone group (P < 0.05). In both groups, the ESR and CRP gradually declined after the operation and had returned to normal at the last follow-up. There was no significant difference between the two groups (P > 0.05). Compared with preoperative scores, the postoperative VAS scores in both groups decreased significantly (P < 0.05), but the mean postoperative VAS score in the structural iliac bone group was higher than that in the morselized bone group (P > 0.05). No significant difference was observed between the two groups at 3 months or the last follow-up (P > 0.05). Neurological outcomes improved significantly after the surgery in both groups, and no significant difference was observed at the last follow-up (Table 2).

**Table 2:** The postoperative clinical and radiological data of patients in two groups

Measurements	Morselized bone group (43)	Structural iliac bone group (39)	P-value
Operative time (min)	144.3 ± 23.6	215.3 ± 32.8	0.008
Blood loss (ml)	337.2 ± 105.2	507.6 ± 167.6	0.003
Hospital stay (d)	11.7 ± 2.1	14.5 ± 2.9	0.001
ESR			
Preoperative	43.3 ± 11.8	54.3 ± 12.5	0.553
Postoperative	21.2 ± 8.0*	19.7 ± 8.1*	0.898
Final follow-up	11.0 ± 4.3*	11.0 ± 4.6*	0.874
CRP			
Preoperative	35.6 ± 14.5	35.7 ± 15.8	0.724
Postoperative	13.2 ± 5.3*	17.4 ± 5.3*	0.854
Final follow-up	7.3 ± 2.3*	6.6 ± 2.6*	0.536
VAS			
Preoperative	6.9 ± 0.9	6.8 ± 0.9	0.903
Postoperative	2.8 ± 0.8*	4.1 ± 0.9*	0.352
3 months follow-up	3.0 ± 0.7*	2.9 ± 0.6*	0.862
Final follow-up	2.3 ± 0.9*	2.4 ± 0.8*	0.417
Cobb (°)			
Preoperative	3.1 ± 6.8	3.1 ± 5.2	0.136
Postoperative	13.4 ± 4.8*	12.6 ± 4.7*	0.99
Final follow-up	10.9 ± 4.0*	11.1 ± 4.4*	0.500
Bone graft fusion time (m)	5.1 ± 1.7	4.7 ± 1.6	0.857
Fusion rate	95% (41/43)	97% (38/39)	0.617
ASIA scores			
C	0	0	0.502
D	1	2	
E	42	37	
Complications	4(9.3%)	10 (25.1%)	0.051

^*^Compared with preoperative (P < 0.05)

### Radiological outcomes

Cohen kappa’s values of interobserver reliability of images parameters was 0.82, therefore we took the average value of the measurements of two senior spinal surgeons as the final value.The postoperative local Cobb angle increased in both groups compared with the preoperative value and decreased slightly at the final follow-up. However, there was no significant difference between the two groups (P > 0.05). Bone fusion was achieved in 41 patients (95%) in the morselized bone group and 38 patients (97%) in the structural iliac bone group, which was not a significant difference (P > 0.05). The mean duration of bony fusion in both groups was not significantly different (P > 0.05).

### Complications

In this study, 4 patients (9.3%) in the morselized bone group had complications, including 1 case of postoperative nerve root irritation, 2 cases of fusion failure, and 1 case of sinus tract formation. In comparison, 10 patients (25.1%) in the structural iliac bone group had complications, including 2 cases of intraoperative dural sac tears, 3 cases of postoperative nerve root irritation, 1 case of fusion failure, and 4 cases of complications at donor sites.

## Discussion

The goal of lumbar spinal tuberculosis surgery is to radically debride lesions, relieve spinal cord compression, restore spinal stability and improve the quality of life of patients [1–3]. A single one-stage anterior approach with interbody fusion has been successfully applied, to the clinic and this method has the advantage of allowing anterior direct decompression and instrumentation. However, anterior surgery may provide insufficient biomechanical stability for the spine, especially for patients with osteoporosis, and it is common to find residual kyphosis or hardware failure at the end of treatment [6, 7]. Thus, the use of a combination of anterior debridement and posterior fixation was implemented, which helped to control the disease early, provide early fusion, and maintain the correction of the kyphosis [8, 9]. However, combination procedures are associated with a large amount of surgical trauma and more postoperative complications.

In recent years, many studies on the treatment of spinal tuberculosis via a single-stage posterior approach have reported good clinical efficacy [5–7]. Radical debridement, bone grafting and internal fixation were performed with one incision, simplifying the operation and greatly reducing the surgical trauma compared with anterior surgery or combined posterior and anterior surgery. Zhang et al. [6] treated thoracolumbar tuberculosis via one-stage posterior debridement, fusion, and pedicle screw fixation and reported that all patients achieved bone fusion within 10 months, without recurrence during a 4-year follow-up. Subsequently, Hassan et al. [16] compared one-stage anterior and posterior surgeries to treat thoracolumbar tuberculosis. The results showed that both the anterior and posterior approaches successfully treated thoracolumbar tuberculosis. These results indicated that posterior-only surgery is feasible and effective for treating thoracolumbar tuberculosis. However, there is also a risk of incomplete lesion clearance, especially for patients with multisegment tuberculosis. Therefore, most authors [5–7, 16] agree that the indications for single posterior surgery include the following: (1) the tuberculosis focus mainly involves the intervertebral space, and bony destruction is present in less than 50% percent of the vertebrae height; (2) the lesion scope does not exceed 2 levels; and (3) the lesion destruction and psoas abscess are mainly on one side and mild on the other side.

Although posterior-only surgery for lumbar tuberculosis has achieved satisfactory clinical results, reconstruction of bony defects after debridement remains a major challenge. Structure iliac bone grafts are the most common method used in spinal tuberculosis surgery. However, morselized bone grafts have also been used to treat spinal tuberculosis with satisfactory results. The difference in clinical and radiographic results between the structural iliac and morselized bone graft methods remains unclear in posterior surgery for lumbar tuberculosis.

In the present study, we compared the clinical efficacy and radiological outcomes of using morselized bone and structural iliac bone to treat lumbar tuberculosis via a posterior-only approach. Compared with the structural iliac bone group, the morselized bone group had the benefit of a shorter operation time and less blood loss. Liu et al. [7] reported similar results using morselized bone as graft material in the surgical treatment of thoracic and lumbar spinal tuberculosis. The reason may be that surgeons must perform multiple tedious procedures to harvest structural bone from a patient’s iliac crest [17]. Moreover, the iliac block must be reshaped, and usually several attempts at insertion must be made because it is difficult to implant a larger size iliac block to reconstruct anterior column defects with a posterior-only approach [18]. In addition, structural iliac bone makes greater demands on the bone graft bed, extensively sclerosed bones need to be removed, and some patients also need to have the pedicles of the lesion segment removed to successfully implant the iliac block [6, 12]. In contrast, local morselized bone is harvested from the vertebral lamina, spinous process, and articular process, and it is easy to prepare and implant, as in TLIF and PLIF surgery, a familiar operative procedure for all spinal surgeons [12, 18]. It does not require extensive intraoperative exposure, shortening the operation time and reducing bleeding. Moreover, the hospital stay in the structural iliac bone group was longer than that in the morselized bone group. We believe that the main reasons are the greater surgical trauma and incidence of postoperative complications in the structural iliac bone group.

The ultimate goal of bone grafting is to achieve bone fusion and long-term segmental stability. There was a lack of consensus on how to evaluate segmental fusion. Although different methods have been reported, the major criterion used to assess fusion is the presence of bone connections between adjacent vertebrae. Proietti et al. [19] believed that it was difficult to evaluate fusion and microinstability, and facet fusion would help in defining the success rate in spinal fusion surgeries as an indirect evaluation method. After intervertebral fusion, spontaneous facet fusion (SFF) may occur, and the fusion rate increases over time. The incidence of SFF showed a large difference (ranging from 19.0 to 68.9%) and is affected by many factors, such as the patient’s age, body mass index, and the presence of arthritis preoperatively [20]. The advantages of SFF include increased spine stability and prevention of internal fixation failure when intervertebral fusion fails. However, there is still controversy about the effect of SFF on the clinical symptoms of patients. The majority believe that SFF does not affect the improvement of clinical symptoms [21, 22]. Interestingly, Proietti et al. [19] divided the surgical segments into immobilization and nonimmobilization groups according to the presence of SFF and found that patients with immobilization had significantly improvements in VAS scores for back pain and the lower limbs and in the ODI compared with the nonimmobilization group.

In our study, the intervertebral fusion status was mainly assessed, while SFF was not careful observated. Based on our observations, both graft methods achieved a good fusion rate that was similar to the outcomes of previous studies, and there was no significant difference in loss of the kyphosis angle between the two groups. The possible reasons are as follows: (1) the majority of internal fixations involved 2 normal vertebral bodies above and below the infected vertebra, which can improve early enough mechanical stability, facilitate early fusion and maintain the correction of kyphosis; (2) implanted morselized bone or iliac bone has very good bone conduction, bone induction, and osteogenic properties, which is more beneficial in promoting early fusion; and (3) early use of a rigid brace contributes to early ambulation and reduces the probability of correction loss and internal fixation failure. Undoubtedly, apart from excellent bone graft materials, complete excision of the lesion and effective anti-tuberculosis drugs played a vital role in successful fusion in patients with spinal tuberculosis.

In the study by Liu et al. [7], the perioperative complication rate was 9.1% (2/22), while in Du’s study [11], the complication rate of posterior surgery for thoracolumbar tuberculosis was 30% (19/61). In this study, 14 cases (17%) had complications, including 4 cases in the morselized bone group (9%) and 10 cases in the structural iliac bone group (25%). The major postoperative complications were intraoperative dural sac tears, postoperative nerve root irritation, and complications at donor sites. We found that the incidence rates of dural sac tears and nerve root irritation were significantly higher in the structural iliac bone group than in the morselized bone group, which may be associated with the intraoperative implantation of the iliac bone. During single-stage posterior surgery for lumbar spine tuberculosis, it is relatively difficult to implant an iliac block due to the presence of lumbar nerve roots [17]. To successfully implant the large-sized bone block, the nerve roots and dural sac of the affected lumbar level were often retracted to make room for the operative procedure. Excessive pulling of the nerve root and dural sac during the implantation of bone grafts may result in postoperative nerve root oedema, causing postoperative pain and numbness in the area innervated by the nerve root [12]. If the bone block is trimmed with an unsmooth surface frequently, it can easily tear the dural sac during implantation [23]. In addition, complications at the donor sites in the structural iliac bone group were higher than those in the morselized bone group, which may be related to harvesting iliac bone from structural iliac bone. Complications at donor sites have been reported in up to 40% of cases [24]. In contrast, using morselized bone as the bone graft material can prevent such iatrogenic injury.

Although satisfactory outcomes were obtained, there are several limitations to this study. First, it was a retrospective study, and the choice of a one-stage posterior approach was mainly based on the surgeon’s experience, which may affect the clinical outcomes between the two groups. Second, only a small sample size and short-term follow-up data were available for both groups. Thus, we believe that a randomized control study with a larger sample size and long-term follow-up is needed to confirm the significance of this study.

## Conclusion

In summary, the two grafting techniques may achieve comparable clinical outcomes in the treatment of lumbar spinal tuberculosis. Compared with structural iliac bone grafts, the morselized bone grafts may have the advantage of involving a short operation time and less blood loss and that was more beneficial in reducing postoperative complications. However, this study was a single-centre retrospective study with a small sample size and a short follow-up time. The above conclusions require further follow-up.

## Data Availability

The datasets supporting the conclusions of this article are included within the article. The raw data can be requested from the corresponding author on reasonable request.

## Abbreviations

VAS: Visual analogue scale
ESR: Erythrocytic sedimentation rate
CRP: C-reactive protein
ASA: American Standards Association
SFF: Spontaneous facet fusion
